# Mitochondrial Thioredoxin System as a Modulator of Cyclophilin D Redox State

**DOI:** 10.1038/srep23071

**Published:** 2016-03-15

**Authors:** Alessandra Folda, Anna Citta, Valeria Scalcon, Tito Calì, Francesco Zonta, Guido Scutari, Alberto Bindoli, Maria Pia Rigobello

**Affiliations:** 1 Department of Biomedical Sciences, University of Padova, via Ugo Bassi 58/b, 35131 Padova, Italy; 2 Institute of Neuroscience (CNR), viale G. Colombo 3, 35131 Padova, Italy; 3 Shangai Institute for Advanced Immunochemical Studies (SIAIS), ShanghaiTech University, No. 99 Haike Road, Pudong, Shanghai 201210, China

## Abstract

The mitochondrial thioredoxin system (NADPH, thioredoxin reductase, thioredoxin) is a major redox regulator. Here we have investigated the redox correlation between this system and the mitochondrial enzyme cyclophilin D. The peptidyl prolyl *cis-trans* isomerase activity of cyclophilin D was stimulated by the thioredoxin system, while it was decreased by cyclosporin A and the thioredoxin reductase inhibitor auranofin. The redox state of cyclophilin D, thioredoxin 1 and 2 and peroxiredoxin 3 was measured in isolated rat heart mitochondria and in tumor cell lines (CEM-R and HeLa) by redox Western blot analysis upon inhibition of thioredoxin reductase with auranofin, arsenic trioxide, 1-chloro-2,4-dinitrobenzene or after treatment with hydrogen peroxide. A concomitant oxidation of thioredoxin, peroxiredoxin and cyclophilin D was observed, suggesting a redox communication between the thioredoxin system and cyclophilin. This correlation was further confirmed by i) co-immunoprecipitation assay of cyclophilin D with thioredoxin 2 and peroxiredoxin 3, ii) molecular modeling and iii) depleting thioredoxin reductase by siRNA. We conclude that the mitochondrial thioredoxin system controls the redox state of cyclophilin D which, in turn, may act as a regulator of several processes including ROS production and pro-apoptotic factors release.

Cyclophilins are a family of peptidyl prolyl isomerases able to catalyze the *cis-trans* isomerization of the peptidyl prolyl bonds (PPIase activity)^1^. CypD, the mitochondrial isoform of the cyclophilin family, is involved in the regulation of the mitochondrial permeability transition pore^2^. Accordingly, mitochondria from CypD^−/−^ mice, are more resistant to Ca^2+^ dependent pore opening or oxidative stress^3^,^4^ compared to the wild type. However, cyclophilins also act as protective factors against oxidative stress since cardiomyocytes lacking CypA are more susceptible to *tert*-butylhydroperoxide treatment^5^. Furthermore, overexpression of CypD protects HEK293 cells from oxidative stress due to *tert*-butylhydroperoxide exposure as it is apparent from the longer maintenance of the mitochondrial membrane potential^6^. Lastly, oxygen stress has been shown to increase the expression of cyclophilin 18 in rabbit blastocysts^7^.

Cyclophilins are endowed with redox properties and they are able to interact with both peroxiredoxin and thioredoxin. Human T cell cyclophilin 18 was shown to bind to the thiol-specific antioxidant protein Aop1 (Prx3) and stimulate its activity^8^. Prx2 can be reduced by human CypA in a cyclosporin A (CsA)-independent process involving Cys-115 and Cys-161^9^. Chloroplast cyclophilin was shown to be inactive in its oxidized form and reactivated by thioredoxin-m, suggesting that cyclophilin is a definite target of Trx^10^. Indeed, peptide mass fingerprint analysis on plant mitochondria identified a peptidyl prolyl *cis-trans* isomerase as a target candidate of Trx^11^. Therefore, in the cell, the ability of Trx to reduce cyclophilin indicates the occurrence of an electron flux from Trx to cyclophilin also involving peroxiredoxin^9^,^10^.

Several observations indicate that cyclophilins are also sensitive to redox conditions. For instance, a ROS-dependent mitochondrial permeability transition associated with increased CypD levels and oxidation has been shown to occur in fibroblasts from patients with X-linked adrenoleukodistrophy and, notably, treatment with both cyclosporin A and N-acetylcysteine^12^ prevented such mitochondrial alterations. According to Linard *et al.*^13^, CypD oxidation affects its conformation and activity suggesting that it could potentially act as a mitochondrial redox sensor. In particular, oxidized CypD could induce mitochondrial permeability transition leading to cell necrosis^13^. The conserved Cys-157 and Cys-203 of CypD act as critical redox-sensitive residues and appear crucial for the activation of the mitochondrial permeability transition pore^13^. The fact that mouse liver mitochondria expressing the CypD mutant Cys203Ser were insensitive to Ca^2+^-induced swelling^14^ and that Cys-203 nitrosylation reduces pore opening in mouse embryonic fibroblasts^14^ further highlights the critical role played by the Cys-203 residue of CypD in inducing mitochondrial permeability transition, possibly through the formation of a disulfide bond with Cys-157^13^.

The thioredoxin system, comprising NADPH, thioredoxin reductase (TrxR) and thioredoxin (Trx), acts by transferring electrons from NADPH to Trx, which, in its reduced form, works as an electron donor for peroxiredoxin (Prx) that, in turn, reduces hydrogen peroxide to water^15^. Thioredoxin is also an important reducing agent for oxidized proteins. Both cytosolic and mitochondrial thioredoxin reductase isoforms possess a C-terminal active site containing a selenocysteine residue with a low pKa^16^, therefore endowed with enhanced nucleophilicity. Consequently, many compounds, including anticancer agents, are considered as effective targets of TrxR^17^,^18^ and, in particular, gold compounds are potent inhibitors of TrxR acting in the nanomolar range^19^,^20^,^21^.

In the present paper we show that both in cancer cells and in isolated mitochondria, TrxR inhibition leads to a change in the redox state of CypD towards a more oxidized form that is potentially responsible for several effects including ROS production and enzyme activity modulation.

## Results

### The thioredoxin system reduces CypD and increases its isomerase activity

**Figure 1:**
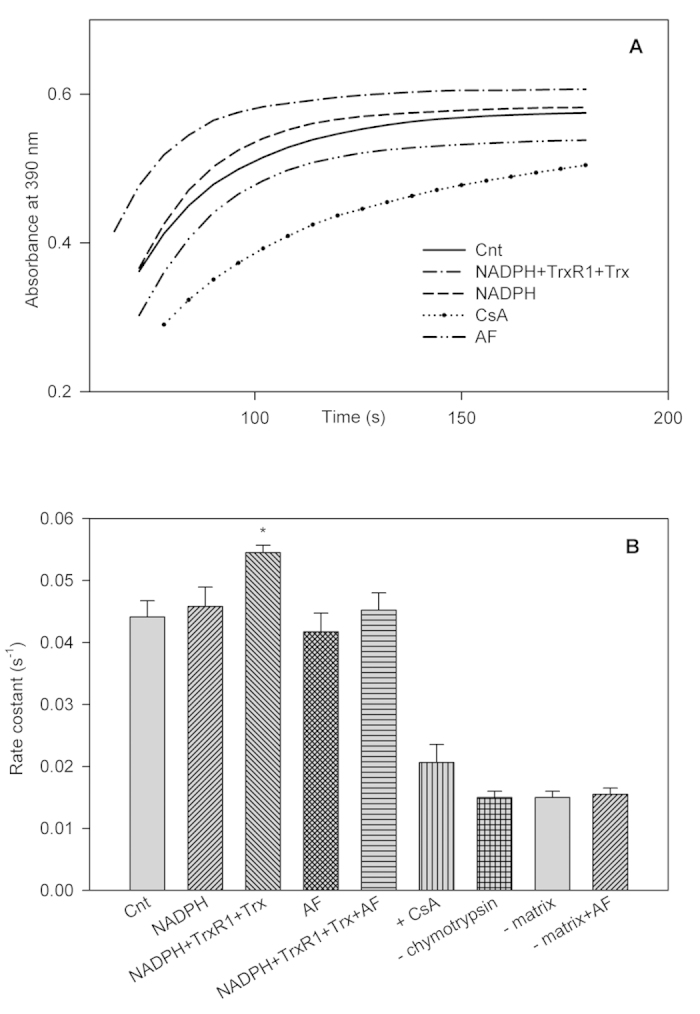
PPIase activity of CypD in the mitochondrial matrix. Mitochondrial matrix (30 μg proteins) was preincubated in 100 mM NaCl, 50 mM HEPES/Tris (pH 8.0) at 25 °C for 15 min in the presence, where indicated, of 300 μM NADPH, 0.4 μM TrxR1, 3 μM Trx from E. coli or 1 μM auranofin (AF). PPIase activity was estimated by a coupled assay utilizing α-chymotrypsin as described under Methods. (**A**) time course of PPIase activity in different experimental conditions and (**B**) first-order rate constants (s^-1^) indicated in the ordinate axis (*p < 0.01) and corresponding to reactions obtained in conditions similar to those of the curves reported in (**A**). Mean ± SD, n = 5 are shown.

Figure 1 shows the PPIase activity measured as absorbance increase of *p*-nitroaniline in a heart mitochondrial matrix fraction incubated under different experimental conditions, while Fig. 1B reports the first order rate constants. Reducing conditions, such as the presence of the complete thioredoxin system (NADPH, TrxR and Trx), stimulated the rate and extent of PPIase activity. NADPH alone had hardly any effect while auranofin (AF), at concentrations able to inhibit thioredoxin reductase but ineffective on CypD, completely prevented the stimulatory effect on PPIase activity elicited by the complete thioredoxin system (Fig. 1B). CsA, a specific inhibitor of cyclophilin D, inhibits PPIase activity, as already shown by Nguyen *et al.*^14^. Finally, the hydrolytic activity of α-chymotrypsin on the peptide N-succinyl-Ala-Ala-Pro-Phe-*p*-nitroanilide was strongly reduced in the absence of mitochondrial matrix preparation, while AF, at the concentrations used, did not alter α-chymotrypsin activity (Fig. 1B). Also the thiol-oxidizing agent diamide markedly inhibited PPIase activity (data not shown). Therefore, the role of the thioredoxin system in maintaining CypD reduced was clearly apparent as reducing and oxidizing conditions increased and decreased PPIase activity of CypD, respectively.

### Inhibition of the thioredoxin system induces CypD oxidation in isolated mitochondria

**Figure 2:**
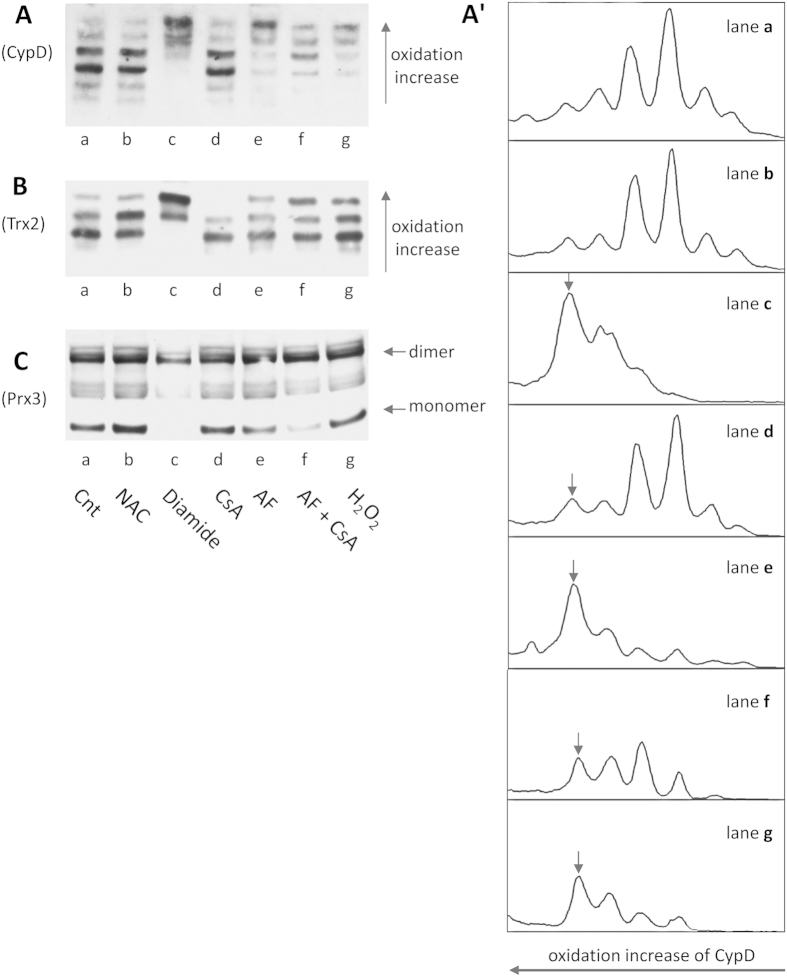
Redox Western blot of CypD (**A**,**A′**), Trx2 (**B**) and Prx3 (**C**) in isolated rat heart mitochondria. Aliquots of 200 μg proteins of rat heart mitochondria were incubated under various conditions at 25 °C for 30 min. (a) control; (b) 2 mM NAC; (c) 2 mM diamide; (d) 1 μM CsA; (e) 5 μM AF; (f) 5 μM AF +1 μM CsA; (g) 2 mM H_2_O_2_. To determine the redox state of CypD and Trx2, samples, derivatized first with 30 mM IAA and then with 10 mM IAM, were subjected to urea-PAGE in non-reducing conditions (**A**,**B**). To analyze the redox state of Prx3, samples, derivatized with 10 mM AIS, were subjected to SDS-PAGE in non-reducing conditions (**C**). (**A′**) densitometric analysis of data reported in panel (**A**) and referred to CypD, and performed using ImageJ software. Bands undergoing oxidation are marked by arrows in (**A′**).

First of all, the redox state of mitochondrial proteins in isolated mitochondria was examined. In Fig. 2 (A,A′), treatment of isolated heart mitochondria with auranofin, a potent inhibitor of thioredoxin reductase^22^,^23^, caused oxidation of CypD as revealed by the appearance in the redox Western blot of a number of bands compatible with the number of thiols present in the enzyme sequence of human CypD^13^. With isolated rat heart mitochondria at least 5 bands were apparent, indicating the presence of 4 thiols potentially subjected to redox transition. Previous observations based on electrophoretic mobility shift or two-dimensional gel electrophoresis showed the oxidation of CypD under specific conditions^13^,^24^. In isolated mitochondria the redox Western blot performed with the “IAA/IAM” method^25^,^26^,^27^ (see also Methods) showed the oxidized bands in the upper part of the gel. In Fig. 2A,A′, lane a (control) shows the redox condition of CypD reflecting the normal redox metabolic state, similar to mitochondria treated with N-acetyl cysteine (NAC) (lane b), while diamide treatment (lane c), illustrates a shift to largely oxidized condition. Incubation of mitochondria with CsA alone did not essentially modify the redox state of CypD (lane d), whereas auranofin showed a strong increase of intensity of the more oxidized bands (lane e). Following TrxR2 inhibition by auranofin, Trx2, turned to a more oxidized form^19^,^20^,^28^,^29^, and this condition is particularly evident when CsA is present together with AF (Fig. 2B, lane f). Regarding oxidation of Trx2 (Fig. 2B), three major bands were detected in accordance with the presence of only two active site cysteines^30^ in Trx2. Also peroxiredoxin, a major substrate of thioredoxin, was found oxidized (dimer) after inhibition of thioredoxin reductase by auranofin (Fig. 2C, lane e) as already demonstrated by Cox *et al.*^31^. The presence of CsA increases the effect of auranofin (Fig. 2C, lane f). In lane g the oxidizing effect of H_2_O_2_ on the examined enzymes is reported. Notably, addition of H_2_O_2_ alone led to an oxidation pattern comparable to that observed with auranofin. Therefore, the oxidation of CypD in mitochondria incubated with either auranofin or H_2_O_2_ appears consistent with that of Trx2 and Prx3, suggesting a redox interplay between the thioredoxin system, peroxiredoxin and CypD.

### ROS production in isolated mitochondria treated with CsA and auranofin

**Figure 3:**
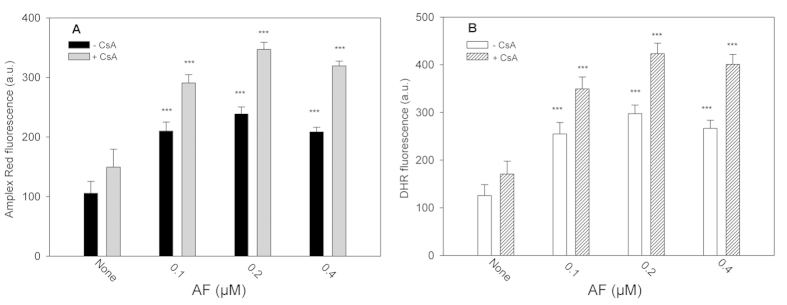
H_2_O_2_ formation in isolated rat heart mitochondria incubated with different concentrations of auranofin in the presence or absence of CsA. Mitochondria (100 μg proteins) were incubated in 100 mM sucrose, 50 mM KCl, 20 mM HEPES/Tris (pH 7.4), 0.5 mM Na,K-phosphate, 5 mM succinate with the indicated concentrations of auranofin and, when present, 5 μM CsA. ROS production was followed using the fluorescent probes Amplex Red (**A**) or DHR (**B**) as described in Methods. The values are the means ± SD of five measurements (***p < 0.001 referred to the respective control).

As ROS, and specifically H_2_O_2_ formation, are indicative of a redox imbalance leading to oxidation of the thiol enzymes involved in mitochondrial redox signaling (Trx2/Prx3/CypD), the effect of CsA on ROS production by isolated mitochondria was examined. Treatment of mitochondria with CsA induced a moderate increase in the amount of H_2_O_2_ as assessed by both Amplex Red oxidation (**A**) and DHR fluorescence decrease (**B**) (Fig. 3). A further increase of H_2_O_2_ level was observed upon inhibition of TrxR2 with a low dose of auranofin, a condition which prevented the removal of hydrogen peroxide as the electron flow to Prx3 was inhibited. The presence of CsA, which binds to CypD, markedly stimulated the production of ROS (about 30%) induced by auranofin in mitochondria.

### Thioredoxin system inhibition determines the oxidation of CypD in tumor cell lines

**Figure 4:**
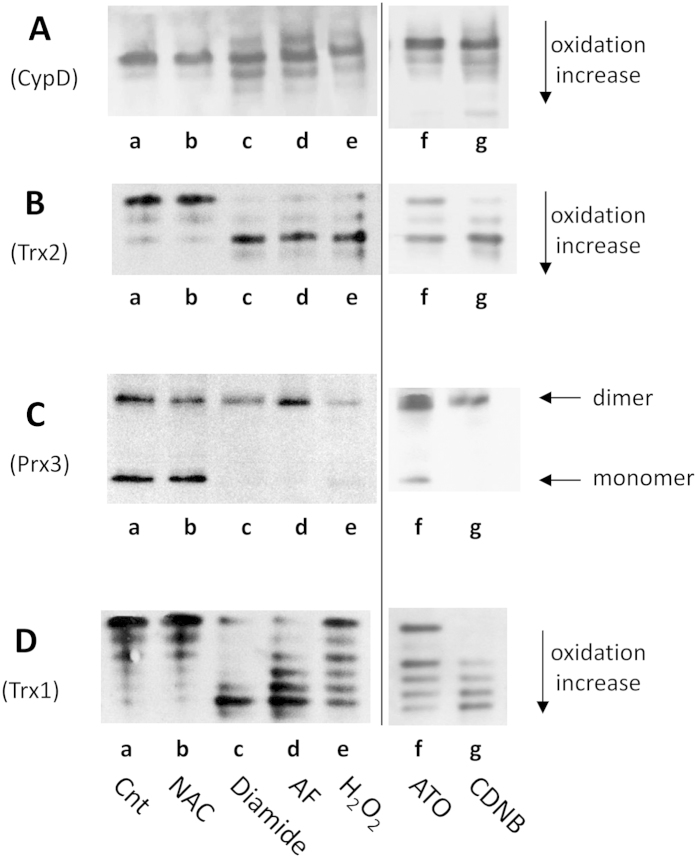
Redox Western blot of CypD, Trx1, Trx2 and Prx3 in CEM-R cells. CEM-R cells (1 × 10^6^), treated for 18 h in various conditions, were derivatized with 10 mM IAM and then with 30 mM IAA for the determination of redox state of CypD, Trx1, Trx2 using urea-PAGE in non-reducing conditions (**A**–**D**). For the estimation of the redox state of Prx3, cell lysates were derivatized with 10 mM AIS and subjected to SDS-PAGE in non-reducing conditions (**C**). (a) control; (b) 2 mM NAC; (c) 2 mM diamide, (d) 3 μM AF; (e) 1 mM H_2_O_2_; (f) 15 μM ATO; (g) 20 μM CDNB.

Alteration of the redox conditions may trigger death of tumor cell lines^20^. Therefore, the redox states of cytosolic and mitochondrial Trxs, Prx3 and CypD in CEM-R cancer cells incubated with NAC, auranofin, arsenic trioxide (ATO), 1-chloro-2,4-dinitrobenzene (CDNB) and the oxidants diamide and hydrogen peroxide were compared. Trxs and CypD were analyzed by using a modified redox Western blot “IAM/IAA”^32^ (see also Methods) in which the upper band represents the fully reduced enzyme, the lowest represent the completely oxidized form, while the intermediate bands represent the formation of disulfides, mixed disulfides with glutathione or protein thiols, or nitrosylated forms. This distribution of different redox states was clearly shown for cytosolic and mitochondrial thioredoxin^30^. As shown in Fig. 4 (lane b), NAC did not substantially change the redox state of CypD, Trxs and Prx 3. In contrast, with the oxidizing agents diamide (lane c), auranofin (lane d) and hydrogen peroxide (lane e), CypD clearly underwent increased oxidation, with bands appearing in the lower part of the gel (Fig. 4A). In similar oxidizing conditions, both cytosolic and mitochondrial thioredoxins underwent to oxidation (Fig. 4B–D) and also Prx3 was in the oxidized form (dimer) (Fig. 4C). In particular, Trx1 exhibited 6 major bands (Fig. 4D) related to the different redox conditions of the five cysteine residues contained in the protein and, at the same time, Trx2 showed three major bands (Fig. 4B)^26^,^30^,^32^. Trx2 was almost completely oxidized indicating that it is more easily oxidized in comparison to its cytosolic isoform^33^. In addition to auranofin, other inhibitors of thioredoxin reductase such as ATO and CDNB^34^,^35^ were tested in CEM-R cells to evaluate the changes of the redox state of CypD, Trxs, and Prx3. As apparent in Fig. 4 (lanes f and g) both ATO and CDNB led to an oxidation of these redox proteins. In particular, CDNB gave results very similar to those obtained with auranofin, while ATO was less effective. Similar results for CypD, Trx2 and Trx1 redox state were found using a different cell line such as HeLa cells (see Supplementary Fig. S1 online). Different concentrations of auranofin or other inhibitors were used in accordance with the amount of thioredoxin reductase present in each cell line. For instance, in HeLa cells the level of thioredoxin reductase is higher than that of other cells^36^. Therefore, in cancer cells, upon induction of oxidizing conditions, there is a concurrent oxidation of Trxs, Prx and CypD. Knockdown of TrxR2 in HeLa cells (see Supplementary Fig. S2 online), although only partial, caused an oxidation of Trx2 and a concomitant moderate oxidation of CypD, again showing a link between mitochondrial thioredoxin system and CypD.

### Interaction of CypD with Trx2 and Prx3: co-immunoprecipitation

**Figure 5:**
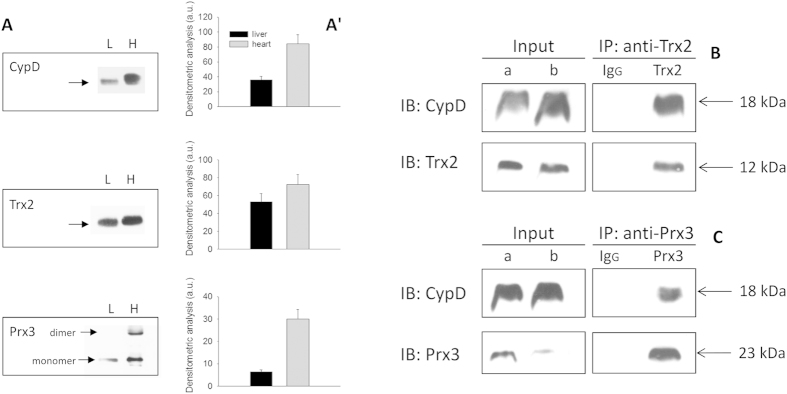
Comparative estimation of CypD, Trx2 and Prx3 in rat liver (L) and heart (H) mitochondrial matrix (**A**,**A′**). Co-immunoprecipitation of CypD with Trx2 (**B**) and Prx3 (**C**). A: comparison of CypD, Trx2 and Prx3 content between liver (L) and heart (H) mitochondrial matrix. Mitochondrial matrix preparations (40 μg protein of each) were analyzed by Western blotting. (**A′**) densitometric analysis of data reported in panel (**A**). (**B**,**C**) pre-reduced rat heart mitochondrial matrix (200 μg protein), was incubated with antibodies anti-Trx2 or anti-Prx3, as described in Methods. The pull down from the immunoprecipitation was separated by SDS-PAGE, blotted and probed with monoclonal anti-CypD. IB: immunoblot. (**B**) co-immunoprecipitation of CypD with Trx2; (**C**) co-immunoprecipitation of CypD with Prx3 with the respective controls. (a) aliquot of heart mitochondrial matrix (10 μg protein); (b) aliquot of supernatant (8 μL) after immunoprecipitation.

In Fig. 5 (A,A′) the comparative estimation in heart and liver mitochondrial matrix of the relative amount of CypD, Trx2 and Prx3 is reported. As shown, the three enzymes are more abundant in the heart than in the liver.

As previously reported, upon inhibition of the thioredoxin system, CypD changes its redox state. Therefore, the interaction of CypD with proteins belonging to the thioredoxin system was studied in rat heart mitochondrial matrix. Fig. 5 (panel B) shows that CypD co-immunoprecipitated with Trx2 when proteins from mitochondrial matrix were immunoprecipitated with a Trx2 antibody and then immunoprobed with a CypD antibody. Similar results were obtained with mitochondrial matrix proteins using a Prx3 co-immunoprecipitation (Fig. 5, panel C). In this case, the co-immunoprecipitation shows that Prx3 and CypD can physiologically interact, consistent with a functional relationship linking these proteins.

In addition, to confirm these results, co-immunoprecipitation was also performed utilizing an anti-CypD antibody to detect Trx2 and Prx3 in the pull down. The results reported in Fig. S3 online, show that there was a coherent connection between the proteins of interest.

### Molecular modelling of the interaction of CypD with Prx3 and Trx2

**Figure 6:**
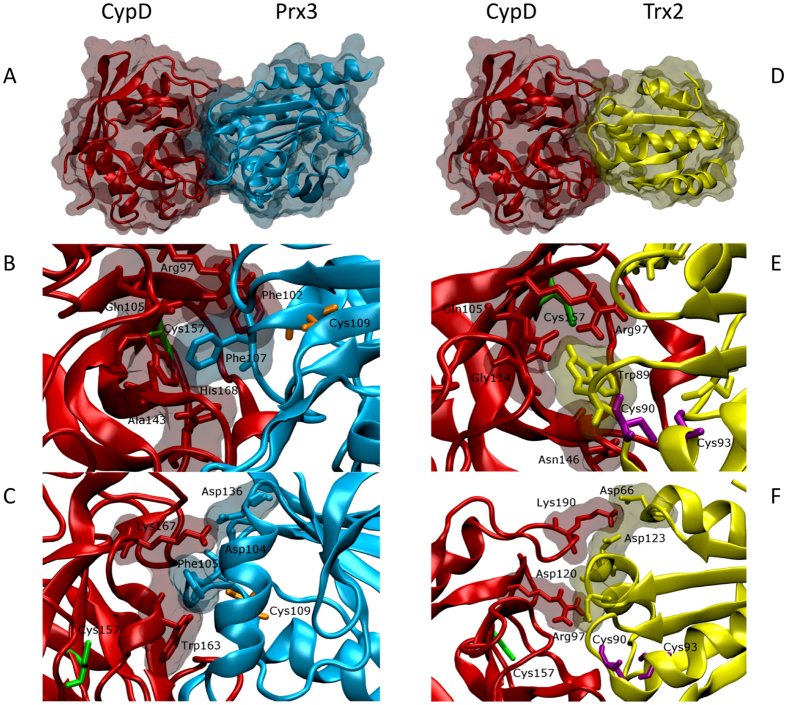
Binding configuration for CypD and Prx3 or Trx2. Panels (**A–C**) show the best predicted binding of CypD and Prx3. Panel (**A**) is the global view of the best binding. Panel (**B**) shows the details of the pocket and one can notice the protrusion of Phe-107 of Prx3 in the hydrophobic pocket of CypD and also are highlighted the residues of CypD that interact with it. Cys-157 of CypD is shown in green while Cys-109 of Prx3 is shown in orange. Panel (**C**) highlights the possible salt bridges formed by the two molecules, in particular those formed by Asp-104 and Asp-136 of Prx3 with Lys-167 of CypD. Panels (**D**–**F**) show the binding of CypD to Trx2. Panel (**D**) is the global view of the best predicted binding. As one can see in Panel (**E**), the interaction of Trp-89 with the hydrophobic pocket is more superficial compared to that of Prx3. The position of Cys-157 of CypD is shown in green, while Cys-90 and Cys-93 of Trx2 are shown in purple. In Panel (**F**), the possible salt bridges that could stabilize the interaction between the two proteins are reported: Arg-97 of CypD with Asp-120 of Trx2, and Lys-190 of CypD with Asp-66 or Asp-123 of Trx2. The amino acids residues are labelled according to full-length proteins sequence.

In order to further explore the nature of the interactions between CypD and Trx2 or Prx3, we performed a molecular docking prediction using ClusPro 2.0 webserver^37^. The results are described below and summarized in legend to Fig. 6. In both cases, the great majority of possible docking configurations involve the hydrophobic binding site of CsA to CypD and aromatic residues in Trx2 and Prx3. These kinds of hydrophobic interactions stabilize the overall structure of protein and protein complexes, and can thus explain in principle the co-immunoprecipitation we have experimentally observed.

### CypD – Prx3 interaction

The great majority of possible predicted interactions between CypD and Prx3 (87%), involves the CsA binding site of CypD. In the most probable binding configuration, Prx3 interacts with the hydrophobic pocket of CypD, in particular with residues Ala-143 and Phe-155, through its Phe-107 (Fig. 6, Panels A and B), and its further stabilized by the formation of the salt bridges showed in Fig. 6 Panel C. Remarkably, this interaction further buries the catalytic Cys-157. The other predicted interactions share similar features, but involve other two phenylalanine residues of Prx3 namely Phe-139 or Phe-222, resulting in different orientation of Prx3 with respect to CypD. Altogether, these results suggest that binding is driven by hydrophobic interaction, and it is facilitated by the presence of exposed hydrophobic side chains in the Prx3 molecule.

### CypD – Trx2 interaction

Similarly to the previously described interaction between CypD and Prx3, also in this case, the great majority of possible bindings (96%) involve the CypD hydrophobic region that is responsible for the binding of CsA. However, in this case, the only accessible aromatic residue is Trp-89, and thus most of the possible binding presents similar mutual orientation between the two molecules. Also in this case, formation of salt bridges help to stabilize the interaction. In the most probable configuration, the proximity of Arg-193 of CypD with Asp-120 of Trx2, and that of Lys-190 of CypD with Asp-66 and Asp-123 of Trx2, suggests that they can be involved in the formation of such bridges as shown in Fig. 6, panel F.

## Discussion

Treatment with auranofin, or other recognized inhibitors of thioredoxin reductase such as ATO and CDNB, induced a change of Trx2, Prx3 and CypD thiol redox state in isolated mitochondria and cultured tumor cells (Figs 2 and 4). A shift towards an oxidized state of CypD was also observed in the presence of the thiol-oxidizing agent diamide, which directly acts on protein dithiols located in close proximity, or with H_2_O_2_ that in the redox signaling processes is considered to function as a second messenger by rapidly reacting with peroxiredoxins which, in turn, may act as sensors of this oxidant^38^. Finally, depletion of TrxR2 with siRNA led to a partial oxidation of CypD concomitant with oxidation of Trx2 (Fig. S2 online). There are several recent reports suggesting an involvement of cyclophilins in redox regulated processes^12^,^24^,^39^,^40^. In X-linked adrenoleukodystrophy, an increased expression and oxidation of CypD in fibroblasts was observed^12^. S-glutathionylation of CypD was also seen in mitochondria isolated from the heart after tachycardia, a condition increasing the oxidation state of the cardiac tissue^24^. Also, cyclophilin A was shown to undergo glutathionylation in cells treated with chloramines and hypochlorous acid, further underlining its role as a redox regulatory protein^40^. Of note, in chloroplasts, cyclophilin 20–3, is reduced and activated by Trx, which, in turn, is maintained reduced by light. Cyclophilin 20–3 can be oxidized by H_2_O_2_/peroxiredoxin B^39^ regulating the cysteine biosynthesis system.

Cyclophilin D plays a major role in controlling the mitochondrial membrane permeability transition pore^41^. The peptidyl prolyl *cis-trans* isomerase activity of CypD in the mitochondrial matrix of liver and heart mitochondria was assessed by Halestrap and Davidson and shown to be sensitive to CsA and correlate with calcium-induced swelling^42^. According to our data, CypD can transduce the redox state to components of the mitochondrial membrane and hence influence its permeability conditions. Auranofin, a well known inhibitor of TrxR^22^,^23^ was previously shown to strongly stimulate the mitochondrial membrane permeability transition^23^. Here we have shown that inhibition of TrxR is responsible for the increased oxidation of CypD. Consequently, the permeability transition pore can be potentially regulated by the redox conditions of CypD which, in its oxidized state, can lead to pore opening while the reverse takes place when CypD is reduced. Of note, CsA which binds to CypD and makes the system similar to the CypD null cells^3^,^4^ does not influence the redox state of CypD. The mitochondrial permeability transition pore is composed of several proteins, but CypD represents the most critical regulatory element^41^. Conditions leading to inhibition of PTP opening are the lack of CypD^3^,^4^, the presence of CsA which prevents the binding of CypD to the mitochondrial components of the PTP located to the inner membrane^2^ and any condition preventing the formation of disulfide groups such as treatment with monothiol reagents^40^, nitrosylation of Cys-203 and mutation of Cys-203 to serine^14^. All these conditions suggest a redox role of CypD in controlling the oxidation state of specific mitochondrial membrane components^43^.

The effect of CsA which, particularly in the presence of AF, leads to a larger production of ROS, suggests that the preservation of membrane integrity and, consequently, of the electron transport carriers, might be responsible of the increased detection of these species (Fig. 3) that may concentrate inside the mitochondrion and subsequently can be released to the cytosol through the aquaporin channel proteins^44^. Furthermore, the donors of reducing equivalents, necessary for ROS production, do not leak out in CsA-treated mitochondria. These results are consistent with previous observations showing that CsA increases ROS formation and lipoperoxidation in cells^45^,^46^,^47^.

The interaction of CypD with Trx2 and Prx3 was also investigated by co-immunoprecipitation and molecular docking analysis. Most cyclophilins are endowed with highly conserved amino acid patch forming the CSA-binding domain (CsA-BD). The opposite side of CsA-BD constitutes the “backface” of CypD which seems to mediate the binding to target proteins^48^. For instance, CypD can dock to phosphate carrier either from the “backface” or from the CsA-binding domain^49^. Further, the interaction between CypD and p53 was shown to be CsA dependent^50^ indicating a docking of CsA-BD to a specific region of p53. We observed that CypD co-immunoprecipitated with Prx3 and Trx2 (Fig. 5 and Fig. S3). To better understand this observation, we also performed an in silico docking simulation to predict the orientation of the CypD binding to the other two proteins. As apparent in Fig. 6, the majority of the possible predicted interactions (87% for Prx3 and 96% for Trx2) show an involvement of the hydrophobic region of CypD, in close proximity to the site of CsA binding. Of note, Cys-90 of Trx2, located in a flexible loop, is close to Cys-157 of CypD.

In conclusion, our data suggest that the mitochondrial thioredoxin system is involved in a specific redox signaling process, where the thiol redox changes can be transmitted to CypD, and presumably further conveyed to several targets such as ATP synthase, ANT, Pi carrier, Bcl2, p53, which are all endowed with redox-sensitive thiols^50^,^51^,^52^,^53^,^54^,^55^. Oxidation of CypD may be due to the action of peroxiredoxin in the presence of hydrogen peroxide, while thioredoxin or other reducing factors may reverse the process. Considering the importance of CypD as the unique recognized factor in the permeability of the mitochondrial membranes, the redox control dependent on the thioredoxin system appears critical for mitochondrial and cellular functioning.

## Methods

### Preparation of mitochondria and mitochondrial matrix

Rat heart mitochondria were isolated by differential centrifugation following the method of Lindenmayer *et al.*^56^. Mitochondrial matrix was obtained by sonication (twice for 30 s each) of a mitochondrial suspension of about 20 mg/ml protein diluted (1:4) with 25 mM Tris/HCl (pH 8.0), followed by centrifugation at 10,000 g. Pellet was discarded and the supernatant centrifuged at 105,000 g for 30 min in order to separate sub-mitochondrial particles from the mitochondrial matrix. The latter was dialyzed overnight against a buffer containing 10 mM Tris/HCl (pH 7.4) and 1 mM EDTA.

Animal (albino rat Wistar) care and relative experimentation were performed in accordance with European and Italian laws (D.L. 26/2014) concerning animal used for scientific purposes. All the protocols were approved by Ethical Committee of University of Padova and all the animals are from an internal animal house authorized by the Ministry of Health (N. 102/2004-A).

### Estimation of peptidyl prolyl cis-trans isomerase (PPIase) activity in rat heart mitochondrial matrix

PPIase activity was estimated essentially as described by Kofron *et al.*^57^ with modifications. Assays were performed at 5.5 °C in 100 mM NaCl and 50 mM HEPES/Tris (pH 8.0). The peptide N-succinyl-Ala-Ala-Pro-Phe-*p*-nitroanilide (Sigma-Aldrich, St. Louis, MO, USA) was dissolved (3 mM) in trifluoroethanol containing 470 mM LiCl, while 2.4 mM α-chymotrypsin (Sigma-Aldrich) was dissolved in 1 mM HCl. Aliquots of mitochondrial matrix (30 μg protein) were preincubated for 15 min in a volume of 50 μl in various conditions as indicated in Fig. 1. The assay was performed in a final volume of 500 μl by the addition, after 5 min of equilibration, of α-chymotrypsin (48 μM, final conc) followed, after 1 min, by the peptide substrate (60 μM, final conc). The reaction was followed spectrophotometrically at 390 nm as absorbance increase of *p*-nitronaniline resulting from the enzymatic cleavage by α-chymotrypsin of the peptide in the *trans* form. The acquired data were fitted to a first-order rate equation in order to obtain the corresponding rate constants (k_obs_, s^−1^)^10^,^13^,^14^.

### Thioredoxin reductase preparation

Highly purified cytosolic thioredoxin reductase (TrxR1) was obtained from rat liver following the procedure of Luthman and Holmgren^58^. Protein content was assayed with the Lowry *et al.* procedure^59^.

### Western blot of mitochondrial matrix for the detection of CypD, Trx2 and Prx3

Aliquots of 40 μg protein of rat liver and heart mitochondrial matrix were loaded onto Bis-Tris Gel NuPAGE (12%) (Novex, Life Technology, Carlsbad, CA, USA) in non-reducing conditions, and then subjected to immunoblot detection with Trx2 polyclonal antibody (Santa Cruz Biotechnology, Inc., Santa Cruz, CA, USA), Prx3 monoclonal antibody (Abfrontier, Young In Frontier co., Seoul, South Korea) and CypD monoclonal antibody (Calbiochem, Merck KGaA, Darmstadt, Germany).

### Determination of ROS production in isolated rat heart mitochondria

Rat heart mitochondria (0.4 mg/ml protein) were incubated at 25 °C in a 96-well plate with 100 mM sucrose, 50 mM KCl, 0.5 mM Na,K-Pi, 20 mM HEPES/Tris (pH 7.4) containing 5 mM succinate and, when indicated, 5 μM CsA. Formation of hydrogen peroxide was detected by using the fluorogenic probe Amplex Red (Invitrogen, Life Technology) at 20 μM in the presence of 11 nM HRP (λ_Ex_ = 544 nm, λ_Em_ = 620 nm) and 5 μM dihydrorhodamine 123 (DHR) (λ_Ex_ = 500 nm, λ_Em_ = 536 nm), using a dual wavelength program with Fluoroskan Ascent FL detector (Labsystems, Finland).

### Cell culture

Leukemic lymphoid CEM-R cells, resistant to vinblastine, were cultured in complete RPMI-1640 medium. Human epithelioid cervix carcinoma HeLa cells were cultured in complete DMEM medium with high glucose.

### Estimation of the redox state of thioredoxins, CypD and Prx3 in mitochondria and tumor cells

The redox state of proteins obtained from isolated mitochondria was estimated following described procedures^25^,^26^,^27^. Briefly, 200 μg protein of freshly prepared mitochondria were incubated in 220 mM mannitol, 70 mM sucrose, 1 mM EDTA, 5 mM HEPES/Tris (pH 7.4), 5 mM glutamate, 5 mM malate in a final volume of 50 μl under different experimental conditions. At the end of incubation time, 100 μl of 11 M urea, 50 mM Tris/HCl (pH 8.3) and 1 mM EDTA containing 30 mM iodoacetic acid (IAA) were added and the reaction was carried out for 30 min at 37 °C, followed by centrifugation. Then, mitochondrial lysates were precipitated by ice-cold acetone-1 M HCl (98:2, v/v). The obtained pellets were washed twice with ice-cold acetone-1 M HCl-H_2_O (98:2:10, v/v/v), resuspended in 60 μL of urea buffer (8 M urea, 50 mM Tris/HCl (pH 8.3) and 1 mM EDTA) with 3.5 mM DTT and incubated for 30 min at 37 °C. Thereafter, samples were treated with 10 mM iodoacetamide (IAM), for 20 min at 37 °C. After protein determination^59^, samples were loaded onto urea-PAGE electrophoresis (7 M urea and 7% acrylamide) in non-reducing conditions and subjected to Western blot detection. With this procedure (“IAA/IAM”) the more oxidized bands appear in the upper part of the blotting gel.

In cells, the protein redox state was estimated by redox Western blot according to the described procedures^25^,^27^ as modified by Du *et al.*^32^. Briefly, cells incubated under different conditions, were treated with 150 μl of 10 mM IAM dissolved in urea lysis buffer (8 M urea, 50 mM Tris/HCl (pH 8.3) and 1 mM EDTA), for 30 min at 37 °C in order to alkylate protein thiol groups. Samples were centrifuged at 14,000 g for 1 min and the resulting supernatant was treated with 10 volumes of cold acetone-1 M HCl (98:2, v/v) and washed with cold acetone-1 M HCl- H_2_O (98:2:10, v/v/v) followed by centrifugation. The pellet was resuspended in 50 μl of urea lysis buffer containing 3.5 mM DTT and incubated for 30 min at 37 °C. After incubation, 30 mM IAA was added and the alkylation reaction carried out for 30 min at 37 °C. After estimation of protein content^59^, samples were then subjected to urea-PAGE electrophoresis (7 M urea and 7% acrylamide) in non-reducing conditions and blotted. With this modified procedure (“IAM/IAA”) the more oxidized bands are found in the lower part of the gel.

For Prx3 redox state estimation the procedure of Stanley *et al.*^60^, with modification, was followed. Cells were centrifuged at 500 g for 5 min, washed with cold PBS and then treated with 1 ml of 10% trichloroacetic acid. Samples, kept at 4 °C for 30 min, centrifuged at 10,000 g for 10 min at 4 °C, were resuspended in 0.5 ml of ice-cold acetone, for 10 min and centrifuged again at 10,000 g for 10 min at room temperature (23–26 °C). After removing the excess of acetone, the pellet was dissolved in 670 mM Tris/HCl (pH 7.5), 2% SDS and 1 mM EDTA containing 10 mM AIS (4-acetamido-4′-((iodoacetyl) amino)stilbene-2,2′-disulfonic acid) (Invitrogen, Life Technology). Derivatization lasted 20 min at room temperature, followed by further 45 min at 37 °C. Samples were loaded, without reducing agents, onto Bis-Tris Gel NuPAGE (12%) and blotted. To assess the redox state of Prx3, a Prx3 monoclonal antibody (Abfrontier, Young In Frontier co.) was used. Rat heart mitochondria (200 μg protein) were processed in the same manner, after incubation under various conditions as reported above.

### Mitochondrial thioredoxin reductase silencing

HeLa cells were seeded at 50% confluence in 6-well plates and transfected with 40–80 nM TXNRD2 siRNA (si GENOME human, Dharmacon, GE Healthcare, Little Chalfont, U.K.) using Attractene (Qiagen, Venlo, The Netherlands) as transfection reagent. Medium was replaced after 6 h and cells were harvested 48 h after transfection. To determine mitochondrial thioredoxin reductase level, cells were lysed in the presence of modified RIPA buffer^29^ and 25 μg proteins were subjected to SDS-PAGE and then to Western blot analysis using monoclonal anti-TrxR2 antibody (SC-166259 Santa Cruz Biotechnology, Inc.). For the loading control anti-GAPDH (Santa Cruz Biotechnology, Inc.) was employed. HeLa cells treated with TXNRD2 siRNA, under the same conditions described above were subjected to IAM/IAA derivatization to determine the redox state of the proteins of interest.

### Co-immunoprecipitation of CypD with Trx2 and Prx3

Rat heart mitochondrial matrix (200 μg protein) was pre-reduced for 30 min at 37 °C in 50 mM Tris/HCl (pH 7.4) in the presence of 0.8 μM TrxR1, 315 μM NADPH and 1 mM EDTA in a final volume of 50 μl. The sample was diluted in 50 mM Tris/HCl (pH 7.4), 50 mM NaCl, 1 mM NaF, 5 mM EDTA and a protease inhibitor cocktail (Complete, Roche, Mannheim, Germany) containing 0.1 mM PMSF. Pre-clearing phase was performed using 20 μl of protein A/G PLUS-Agarose (Santa Cruz Biotechnology, Inc.) for 45 min at 4 °C with stirring. Pre-cleared mitochondrial matrix was centrifuged at 720 g for 5 min and the supernatant was incubated with Trx2 monoclonal antibody (Abfrontier, Young In Frontier co.) or Prx3 monoclonal antibody (Santa Cruz Biotechnology, Inc.) at 4 °C for 2 hours. Slurry of A/G PLUS-Agarose (20μl) was added to the mitochondrial matrix, incubated for 1 h at 4 °C, centrifuged at 720 g for 5 min and washed with ice-cold RIPA buffer modified, and then with 50 mM Tris/HCl (pH 7.4), 1 mM EDTA, 1 mM NaF and a protease inhibitor cocktail (Complete, Roche). The pull down was resuspended in SDS loading buffer containing 100 mM DTT, boiled for 10 min, and then centrifuged at 17,000 g for 1 min. The supernatant was separated by SDS-PAGE (Mini-PROTEAN^®^ TGX, any kD, Bio-Rad Lab. Inc., Hercules, CA, USA), transferred onto a nitrocellulose membrane using Trans-Blot^®^ Turbo Blotting System (Bio-Rad Lab Inc.). Membrane blots were probed with Trx2 polyclonal antibody (Santa Cruz Biotechnology, Inc.), Prx3 monoclonal antibody (Abfrontier, Young In Frontier Co.), CypD monoclonal antibody (Calbiochem, Merck KGaA) and visualized by enhanced chemiluminescence. Mitochondrial matrix (10% of initial volume) was also loaded as an input control. In addition, similar experiment was performed utilizing an anti-CypD antibody to co-immunoprecipitate Trx2 and Prx3, using the same procedure.

### Molecular modelling of the interaction between CypD and Prx3 or Trx2

A molecular docking prediction using ClusPro 2.0 webserver^37^ was utilized. For CypD, Prx3, and Trx2 the protein structures present in PDB (Protein Data Bank), 2bit (human CypD), 1zye (bovine Prx3) and 1uvz (human Trx2) respectively were utilized. For Prx3, 1zye crystal structure that presents more than 88.8% identity with human sequence, was used.

### Statistical Analysis

Statistical analysis was done using GraphPad Instat, GraphPad Software, Inc. All the values are the means ± SD of at least five measurements. Multiple comparisons were made by one-way analysis of variance followed by Tukey-Kramer multiple comparison test.

## Additional Information

**How to cite this article**: Folda, A. *et al.* Mitochondrial Thioredoxin System as a Modulator of Cyclophilin DRedox State. *Sci. Rep.* **6**, 23071; doi: 10.1038/srep23071 (2016).

## Supplementary Material

Supplementary Information
