# Supplementary material for: Mitochondrial Thioredoxin System as a Modulator of Cyclophilin D Redox State
Source: Sci Rep. 2016 Mar 15;6:23071. doi: 10.1038/srep23071 (PMC4791683; doi:10.1038/srep23071)
Supplement: Supplementary Information [file srep23071-s1.pdf]

Supplementary Information

Mitochondrial Thioredoxin System as a Modulator of Cyclophilin D Redox State

Alessandra Folda[a], Anna Citta[a], Valeria Scalcon[b], Tito Calì[a], Francesco Zonta[c], Guido Scutari[a], Alberto Bindoli[b*], Maria Pia Rigobello[a*]

[a]Department of Biomedical Sciences, University of Padova, via Ugo Bassi 58/b, 35131 Padova, Italy

[b]Institute of Neuroscience (CNR), viale G. Colombo 3, 35131, Padova, Italy

[c]Shangai Institute for Advanced Immunochemical Studies (SIAIS), ShanghaiTech University, No. 99 Haike Road, Pudong, Shanghai 201210, China

alessandra.folda.1@unipd.it; anna.citta@unipd.it; valeria.scalcon@studenti.unipd.it;

tito.cali@unipd.it; fzonta@shanghaitech.edu.cn; guido.scutari@unipd.it;

alberto.bindoli@bio.unipd.it; mariapia.rigobello@unipd.it.

Corresponding Author: Maria Pia Rigobello, via Ugo Bassi 58/b, 35131 Padova, Italy.

Phone: +39-049-8276138; e-mail: mariapia.rigobello@unipd.it.

Co-corresponding Author: Alberto Bindoli, viale G. Colombo 3, 35131, Padova, Italy

Phone: +39-049-8276138; e-mail: alberto.bindoli@bio.unipd.it

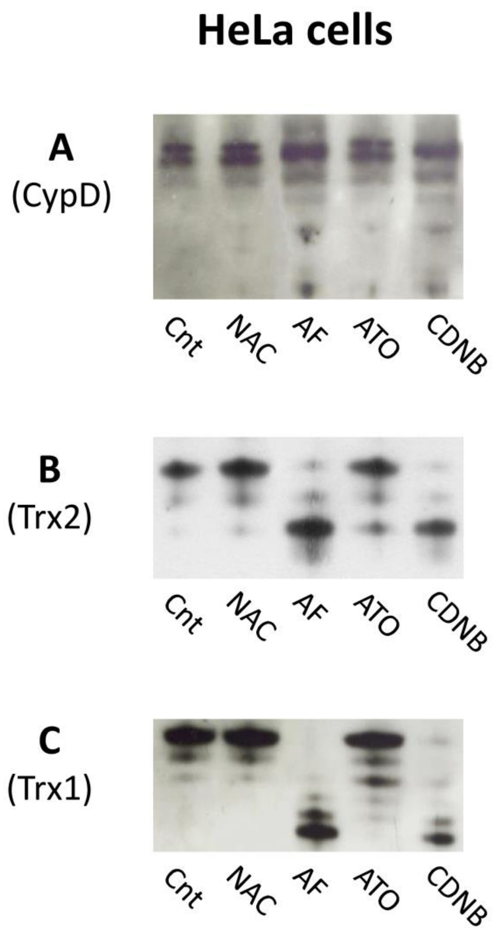

Fig. S1. Redox state of CypD, Trx2 and Trx1 in HeLa cells

A. HeLa cells (5 x 10^5) were treated for 18 h in the following conditions: Cnt, 3 mM NAC, 15 µM AF, 20 µM ATO and 20 µM CDNB. Cells, washed with cold PBS, were rapidly derivatized first with 10 mM IAM and then with 30 mM IAA as described in Methods.

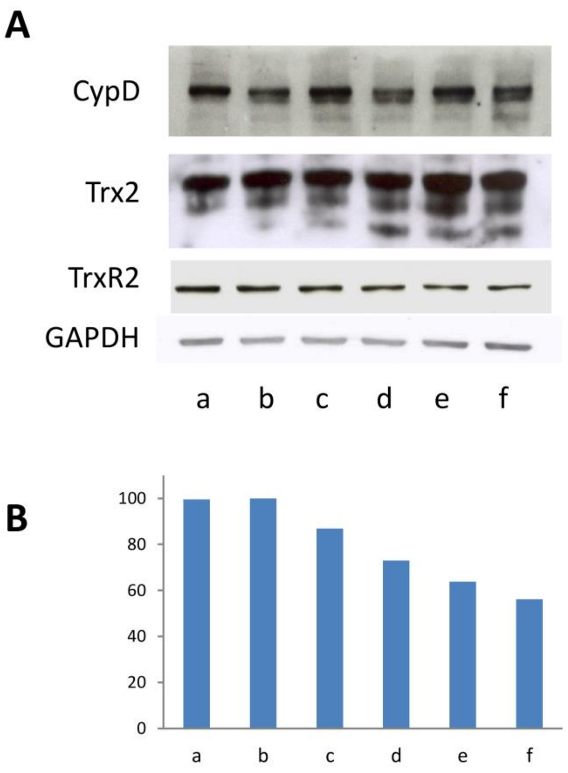

Fig. S2. siRNA silencing of TrxR2 in HeLa cells

HeLa cells were transfected with 40 nM (c) 80 nM (d) of TXNRD2 siRNA (target sequence GAAAGAGAUUCUGCUGUCA) and 40 nM (e) and 80 nM (f) of TXNRD2 siRNA (target sequence GCCGAUCACAUCAUCAUUG). (a) mock-treated control; (b) siControl-non targeting siRNA. Panel A. CypD and Trx2 redox state, TrxR2 protein level and GAPDH as loading control of TrxR2. For CypD and Trx2 redox state detection, HeLa cells were derivatized with IAM/IAA method after siRNA treatment. Panel B. Densitometric analysis of TrxR2 proteins level after treatment with the indicated concentrations of siRNA.

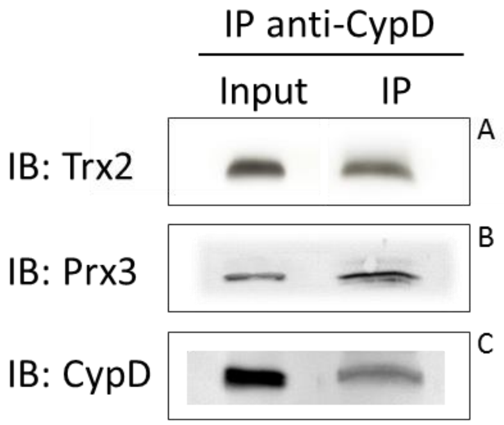

Fig. S3. Co-immunoprecipitation of CypD with Trx2 and Prx3 utilizing antibody anti-CypD.

Pre-reduced rat heart mitochondrial matrix (200 µg protein), was immunoprecipitated with antibody anti-CypD, as described in Methods. The pull down was separated by SDS-PAGE, followed by Western blotting with anti-Trx2 and anti-Prx3 antibodies. IB: immunoblot. A: co-immunoprecipitation of anti-CypD with Trx2; B: co-immunoprecipitation of anti-CypD with Prx3 with the control (C). Input: aliquot of heart mitochondrial matrix (10 µg protein).
